# Dose-Response of Tenapanor in Patients With Hyperphosphatemia Undergoing Hemodialysis in Japan—A Phase 2 Randomized Trial

**DOI:** 10.1016/j.ekir.2021.11.008

**Published:** 2021-11-24

**Authors:** Masaaki Inaba, Yotaro Une, Kazuaki Ikejiri, Hironori Kanda, Masafumi Fukagawa, Tadao Akizawa

**Affiliations:** 1 Ohno Memorial Hospital, Osaka, Japan; 2 R&D Division, Kyowa Kirin Co., Ltd., Tokyo, Japan; 3 Division of Nephrology, Endocrinology, and Metabolism, Department of Internal Medicine, Tokai University School of Medicine, Kanagawa, Japan; 4 Division of Nephrology, Department of Medicine, Showa University School of Medicine, Tokyo, Japan

**Keywords:** chronic kidney disease, hemodialysis, hyperphosphatemia, NHE3 transporter, phosphorus absorption inhibition, tenapanor

## Abstract

**Introduction:**

Simplified, but effective, hyperphosphatemia treatments with novel mechanisms of action, tolerable safety profiles, and low pill burden are needed for patients undergoing hemodialysis. Tenapanor is a calcium (Ca)-free, nonmetal, nonpolymeric drug that reduces phosphate absorption by selectively inhibiting intestinal sodium-hydrogen exchanger 3. As the serum phosphorus (P) level-lowering effect of tenapanor has not been evaluated in Japanese patients with hyperphosphatemia undergoing hemodialysis, we evaluated its efficacy and safety in this population.

**Methods:**

This was a multicenter, phase 2, double-blind, placebo-controlled, parallel-group, dose-finding study. Change in serum P level from baseline at week 6 was the primary end point.

**Results:**

Overall, 207 patients were randomized to 5 groups (placebo [*n* = 41] and tenapanor 5-mg taken twice daily [BID] [*n* = 42], 10-mg BID [*n* = 41], 30-mg BID [*n* = 42], and 30-mg BID dose-titration [*n* = 41]) and treated for 6 weeks. Mean changes from baseline at week 6 in serum P level were 0.64, −0.93, −1.36, −1.92, and −1.99 mg/dl in the placebo and tenapanor groups, respectively. Serum P level was significantly decreased from baseline in all tenapanor groups compared with placebo (*P* < 0.001, for each dose). Diarrhea was the most frequent drug-related adverse event (AE) with an incidence of 9.8%, 50.0%, 65.9%, 76.2%, and 65.9% in the respective placebo and tenapanor groups.

**Conclusion:**

In Japanese patients undergoing hemodialysis, tenapanor was found to have a dose-responsive, serum P level-lowering effect. Diarrhea was the most frequent drug-related AE; most cases were mild and generally tolerable. Tenapanor may become a first-in-class therapeutic agent for patients with hyperphosphatemia.

## Graphical abstract

**Figure:**
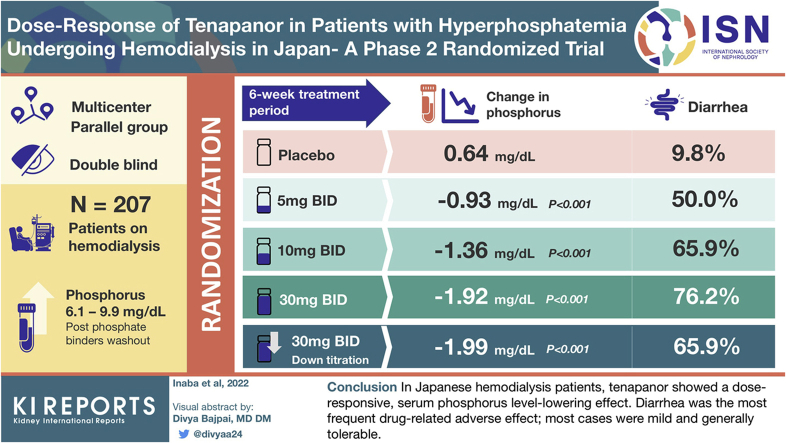

Approximately 334,505 individuals were undergoing long-term dialysis in Japan in 2017,^1^ representing a 1.5% increase compared with 2016.^2^ Furthermore, approximately 97.2% of these individuals were undergoing hemodialysis.^1^ Despite advancements in dialysis technology and treatment for chronic kidney disease (CKD), patients with progressive disease develop complications, including derangements of the bone and mineral metabolism that present as abnormalities in parathyroid hormone (PTH), vitamin D, Ca, and P. These bone and mineral metabolic alterations lead to bone lesions and various diseases^3^^,^^4^ and predispose patients to a high risk of cardiovascular calcifications.^4^^,^^5^ Currently, the syndrome encompassing this spectrum of extraskeletal manifestations and bone lesions is known as CKD-mineral bone disorder.^4^^,^^6^

The development of CKD-mineral bone disorder is associated with poor disease outcomes, increased morbidity, decreased quality of life, and increased cardiovascular mortality.^7^ Increased serum P levels, resulting from alterations in phosphate metabolism and renal function decline as CKD progresses,^8^ are associated with a higher risk of death among patients undergoing hemodialysis.^9^ Hyperphosphatemia is also a well-known risk factor for vascular calcification^10^, ^11^, ^12^; accumulation of P in the body may increase the Ca × P product and lead to calcium phosphate deposits in the vascular walls, heart, conjunctiva, and kidneys.^12^

Given this context, and according to current CKD-mineral bone disorder guidelines, it is essential to control serum P levels in patients with CKD-mineral bone disorder.^13^^,^^14^ If proper management of serum P levels is difficult to achieve by dialysis and dietary intervention, the use of phosphate binders is recommended. Although different types of phosphate binders (e.g., lanthanum carbonate, sucroferric oxyhydroxide, calcium carbonate, ferric citrate hydrate, sevelamer hydrochloride, and bixalomer) are effective in decreasing serum P levels by physically binding to dietary P,^15^ some patients present AEs that may limit treatment with these drugs.^14^^,^^16^ These drugs also tend to have a high pill burden^17^ (i.e., an increased number of pills to be taken regularly), which can lead to poor treatment adherence.^17^^,^^18^

Simplified but effective hyperphosphatemia treatments with novel mechanisms of action distinct from conventional phosphate binders, tolerable safety profiles, and low pill burden are in demand for patients with CKD and hyperphosphatemia undergoing hemodialysis. These much-needed hyperphosphatemia treatments could contribute to improved adherence to therapy and improved serum P levels.

Tenapanor is a novel, Ca-free, nonmetal, nonpolymeric drug that reduces phosphate absorption by selectively inhibiting the intestinal sodium-hydrogen exchanger 3 on the surface of enterocytes.^19^ This results in the suppression of the passive transport of P absorption and reduced serum P. Simultaneously, sodium absorption is inhibited, leading to increased water secretion into the intestinal tract and causing loose stools.^20^ The clinical efficacy of tenapanor has been revealed in a phase 3, randomized, placebo-controlled trial of patients with hyperphosphatemia undergoing maintenance hemodialysis in the United States.^21^ The main drug-related AE was diarrhea, with an incidence of 47.9%. Another key feature of tenapanor is that the tablet is small and the dose consists of 1 tablet BID; thus, the added pill burden is expected to be low. A recent study revealed that adding tenapanor to the phosphate binder regimen of patients undergoing hemodialysis decreased the number of daily phosphate binder tablets necessary to achieve a serum P level within the baseline range of ±0.5 mg/dl.^22^

The serum P level-lowering effect and safety of tenapanor in Japanese patients with hyperphosphatemia undergoing hemodialysis have not been evaluated. This phase 2 study aimed to evaluate the efficacy and safety of tenapanor and determine the clinically recommended dose by comparing changes in serum P levels from baseline and safety outcomes between patients with hyperphosphatemia undergoing hemodialysis receiving tenapanor treatment for 6 weeks and those receiving placebo.

## Methods

### Study Design, Randomization, and Treatment

This was a multicenter, randomized, phase 2, double-blind, placebo-controlled, parallel-group, dose-finding study conducted at 31 sites in Japan from April 2019 to December 2019. The institutional review board at each participating center approved the study protocol and associated documents. The study was conducted in accordance with the Declaration of Helsinki and in compliance with the Law on Drugs, Medical Devices, Good Clinical Practice Ordinance, and its partially revised Ministerial Ordinance. All participants provided informed consent at study enrollment. The trial was registered at ClinicalTrials.gov under the identifier NCT03864458.

**Figure 1:**
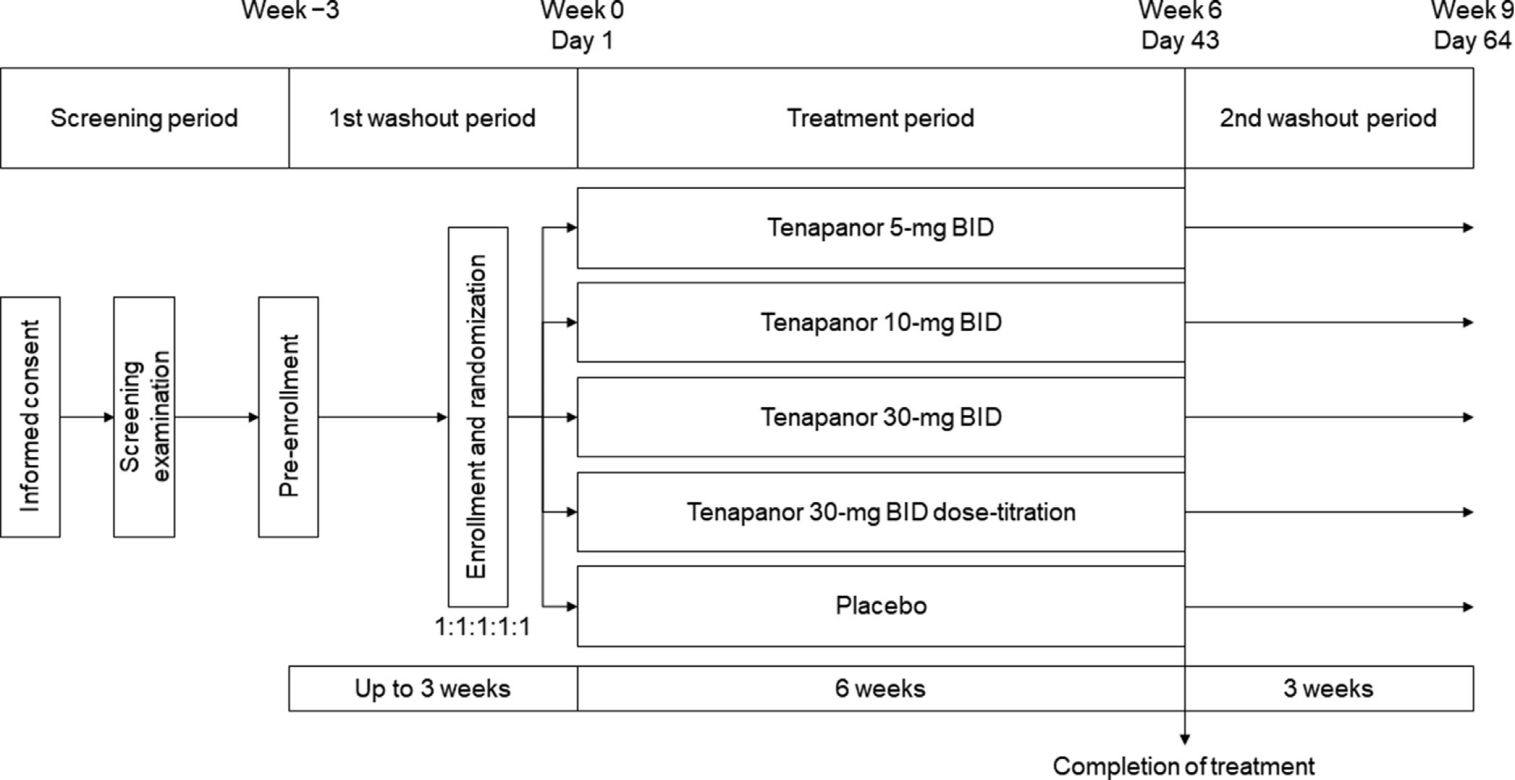
Study design. BID, twice daily.

The study had a screening period (from the date of providing informed consent until pre-enrollment), a first washout period (up to 3 weeks from pre-enrollment until enrollment), a treatment period (6-week treatment period [weeks 0–6]), and a second washout period (3 weeks after completion of the study treatment [weeks 7–9]) (Figure 1).

There were 5 treatment groups, as follows: tenapanor 5-mg BID, 10-mg BID, 30-mg BID, and 30-mg BID dose-titration groups, and a placebo group. The tenapanor dose could be adjusted only for patients in the 30-mg BID dose-titration group. In the tenapanor 30-mg BID dose-titration group, the starting dose was tenapanor 30 mg BID, and it could be down-titrated by the investigator in a stepwise manner (weekly) up to 3 times to 20 mg BID, 10 mg BID, or 5 mg BID. Dose adjustments were performed at the investigator's discretion based on study drug-related gastrointestinal symptoms.

At enrollment, patients were randomly assigned to each treatment group in a 1:1:1:1:1 ratio by an Interactive Web Response System. The allocation factors were serum P level (≥6.1 mg/dl and ≤8.0 mg/dl or ≥8.1 mg/dl and <10.0 mg/dl) and investigative site. Treatment was initiated on day 1 (week 0) of the treatment period, which was the day of the first dialysis after the longest dialysis interval after enrollment. In principle, patients self-administered the study drug (tenapanor or placebo) BID immediately before breakfast and dinner in a double-blind manner for 6 weeks. Patients received 4 bottles of the study drug weekly (1 bottle of 5-mg tablets; 3 bottles of 10-mg tablets) and were instructed to take 1 tablet from each bottle per administration (4 tablets in total). Doses were adjusted using active drug- and placebo-filled bottles according to the direction by the Interactive Web Response System. Patients recorded the dates and times of study drug treatment and whether the tablets were taken as prescribed in a patient diary. Investigators checked patient diaries at each visit. Details regarding prohibited and allowed concomitant medications are provided in the Supplementary Methods.

### Patients

This study targeted patients with hyperphosphatemia undergoing hemodialysis who were being treated with phosphate binders. The main inclusion criteria at pre-enrollment were as follows: patients aged 20 to 80 years; had undergone hemodialysis 3 times per week for ≥12 weeks; had unchanged dialysis conditions (dialysate, dialyzer, frequency of dialysis per week, dialysis duration, blood flow rate, and dialysate and substitution fluid flow rates), excluding dry weight, during the previous 2 weeks; were taking the same regimen of phosphate binders 3 times per day during the previous 4 weeks; were receiving the same regimen of vitamin D or calcimimetics during the previous 4 weeks; and had Kt/V urea ≥1.2 at the most recent test in routine medical practice before the screening examination.

The main exclusion criteria at pre-enrollment were as follows: intact PTH >600 pg/ml before enrollment; diagnosis of irritable bowel disease or diarrhea-predominant irritable bowel syndrome; and gastrointestinal tract surgery, such as gastrectomy or enterectomy (excluding endoscopic resection and cecectomy). Other exclusion criteria are listed in the Supplementary Methods.

For enrollment, patients had to have serum P levels within the range of ≥6.1 and <10.0 mg/dl and increased by ≥1.0 mg/dl during the first washout period. Those with diarrhea or loose stools, defined as Bristol Stool Form Scale (BSFS) score ≥6 and frequency ≥3 for at least 2 days within 1 week before enrollment, were excluded. Changes in dialysis conditions other than dry weight were prohibited from the 2 weeks before the screening examination to the final examination at the end of the study. Patients could not change their diet or partake in new dietary interventions during the study period.

### Study End Points

The primary end point was the change in serum P level from baseline at week 6. The secondary end points were changes in serum phosphorous levels from baseline at each time point, time course of serum P levels, achievement of the target serum P level (serum P level: ≤6.0, ≤5.5 mg/dl), time when the target serum P level was achieved, and changes in Ca × P product and corrected serum Ca levels from baseline at each time point. The exploratory end points were time course of intact fibroblast growth factor (iFGF) 23, intact PTH levels, and bone turnover markers (bone-specific alkaline phosphatase, osteocalcin, total N-terminal propeptide of type I collagen, and tartrate-resistant acid phosphatase 5b).

The safety end points were AEs listed by severity, seriousness, action taken, outcome, and causality. Frequencies were calculated by System Organ Class and Preferred Term using the Medical Dictionary for Regulatory Activities version 22.1. The severity of AEs was judged by the investigator as mild (i.e., signs or symptoms are present but do not interfere with daily activities), moderate (i.e., discomfort that interferes with daily activities or affects clinical status), or severe (i.e., inability to perform daily activities; having a significant impact on clinical status). Changes in laboratory values and vital signs were also evaluated.

### Data Collection

Data were collected in electronic case report forms and patient diaries and included demographics, clinical characteristics, past medical history, primary disease, previous phosphate binder treatment, physical examination, defecation status, dialysis condition, treatment exposure, laboratory tests, vital signs, and 12-lead electrocardiogram. General biochemical tests, serum P level, serum Ca level, and Ca × P were evaluated weekly; intact PTH was evaluated every 2 weeks; and iFGF23, vitamin D-related parameters, and bone turnover markers were evaluated at the pretest and weeks 0 and 6. Changes in average BSFS scores and stool frequency per week by group were also evaluated.

### Statistical Analysis

The target sample size for the study was determined by reference to results from a study conducted outside of Japan.^23^ On the basis of that study,^23^ the effect of tenapanor 30 mg on placebo was ≥1.5 mg/dl. The SD was 2.0 mg/dl for the changes in serum P levels from baseline levels at week 6. Thus, 40 subjects per group were required to provide a 90% power to detect differences using a 4-arm Williams test with a 1-sided significance level of 2.5%. Including the 30-mg BID dose-titration group, the total number of subjects receiving study treatment in this study was set to 200.

The modified intent-to-treat population was used for efficacy analyses. It included all subjects randomized to treatment who had received the study drug and had available serum P level measurements since the start of the treatment. The safety analysis set included all subjects who were eligible for enrollment and who had received the study drug during the treatment period.

For each treatment group, categorical data were summarized using frequencies and percentages; continuous data were summarized using the number of patients, mean, SD, minimum, median, and maximum. For the primary end point, a Williams test was conducted to determine differences from placebo. If the serum P level at week 6 was missing, the serum P level at the last measurement time point was imputed by the last observation carried forward method. A 1-sided *P* value of 2.5% was used for the Williams test. A mixed-effects model for repeated measures was used for sensitivity analysis. The analysis using the mixed-effects model for repeated measures was conducted with change in serum P level from baseline as a response variable; treatment groups as an explanatory variable; and time points, serum P levels on day 1, and interaction of treatment groups with time points as covariates. A *t* test was performed to compare the tenapanor 30-mg BID dose-titration and placebo groups with the tenapanor 30-mg BID dose-titration and tenapanor 30-mg BID groups. SAS version 9.4 (SAS Institute Inc., Cary, NC) was used for statistical analyses.

## Results

### Patient Disposition and Background Characteristics

**Figure 2:**
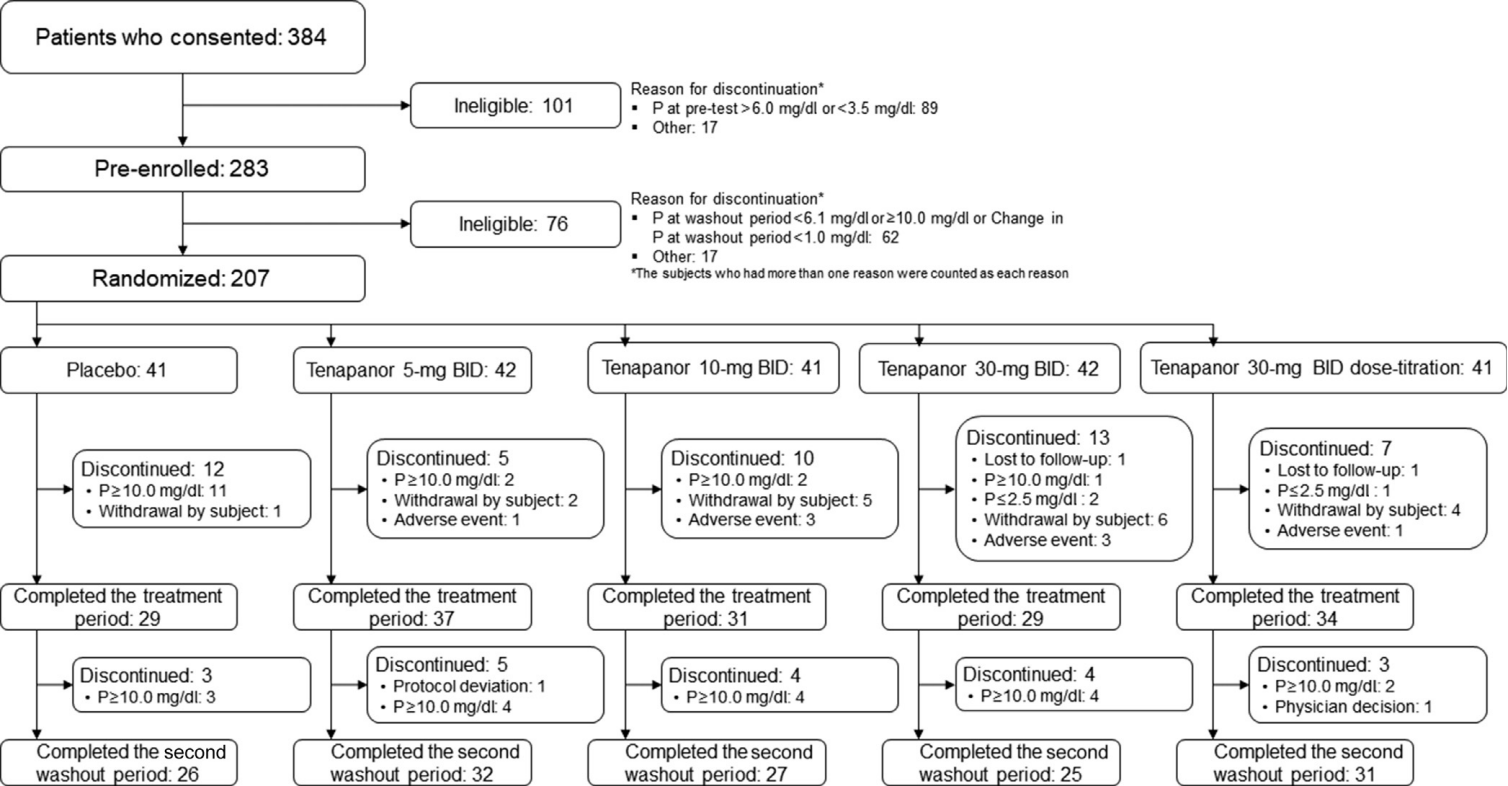
Patient disposition. BID, twice daily; P, phosphorus.

Of the 207 patients enrolled, 41, 42, 41, 42, and 41 patients were randomly assigned to the placebo and tenapanor 5-mg BID, 10-mg BID, and 30-mg BID groups and the 30-mg BID dose-titration group, respectively. All 207 patients received at least 1 dose of the study drug and were included in the modified intent-to-treat and safety analysis sets. A total of 47 patients discontinued during the treatment period: 12, 5, 10, 13, and 7 patients, respectively. The main reason for discontinuation in the placebo group was serum P level ≥10.0 mg/dl in 11 patients. In contrast, the main reasons for discontinuation in the tenapanor 5-mg BID, 10-mg BID, 30-mg BID, and 30-mg BID dose-titration groups were patient withdrawal (2, 5, 6, and 4 patients, respectively) and AEs (1, 3, 3, and 1 patients, respectively). During the second washout period, 19 patients discontinued treatment: 3, 5, 4, 4, and 3, respectively. The main reason for discontinuation in the placebo and tenapanor groups was serum P level ≥ 10.0 mg/dl (Figure 2).

Table 1 illustrates patient baseline characteristics by group. Across the placebo and all the tenapanor groups, >60% of patients were male, the mean age was between 61 and 65 years, and the body mass index was between 23 and 24 kg/m^2^. Overall, the most common primary diseases were diabetic nephropathy and chronic glomerulonephritis, followed by nephrosclerosis. The most often prescribed phosphate binders before the first washout period were lanthanum carbonate and calcium carbonate (both prescribed in >50% of patients in each group). At baseline, the mean (SD) serum P levels in the placebo and tenapanor 5-mg BID, 10-mg BID, 30-mg BID, and 30-mg BID dose-titration groups were 7.6 (1.3) mg/dl, 7.5 (1.1) mg/dl, 8.1 (1.1) mg/dl, 7.7 (1.4) mg/dl, and 7.4 (1.1) mg/dl, respectively. Overall, the treatment groups were well balanced. The dose breakdown in the tenapanor 30-mg BID dose-titration group at week 6 was as follows: 30 mg, 25 of 35 (71.4%); 20 mg, 4 of 35 (11.4%); 10 mg, 5 of 35 (14.3%); and 5 mg, 1 of 35 (2.9%).

**Table 1:** Baseline demographic and clinical characteristics of patients by treatment group

Parameter, unit	Placebo	Tenapanor (BID)			
		5 mg	10 mg	30 mg	30 mg dose-titration
*N*	41	42	41	42	41
Sex					
Female	13 (31.7)	16 (38.1)	13 (31.7)	12 (28.6)	14 (34.1)
Male	28 (68.3)	26 (61.9)	28 (68.3)	30 (71.4)	27 (65.9)
Age, yr					
Mean (SD)	63.9 (10.5)	63.6 (10.4)	65.4 (8.8)	63.1 (9.2)	61.7 (10.0)
Median (min–max)	67.0 (40–78)	66.0 (39–79)	67.0 (40–77)	64.0 (40–77)	64.0 (35–78)
<65	18 (43.9)	18 (42.9)	18 (43.9)	22 (52.4)	22 (53.7)
≥65	23 (56.1)	24 (57.1)	23 (56.1)	20 (47.6)	19 (46.3)
Height, cm					
Mean (SD)	161.3 (7.5)	162.0 (9.9)	162.1 (9.5)	162.4 (8.8)	163.0 (8.7)
Weight at d 1 before dialysis (kg)	*n* = 41	*n* = 42	*n* = 41	*n* = 42	*n* = 40
Mean (SD)	61.7 (10.7)	63.1 (12.9)	61.0 (10.6)	65.3 (13.3)	65.4 (12.0)
Weight at d 1 after dialysis (kg)	*n* = 41	*n* = 42	*n* = 41	*n* = 42	*n* = 40
Mean (SD)	59.0 (10.3)	60.3 (12.7)	58.3 (10.3)	62.5 (12.6)	62.6 (11.5)
Body mass index, kg/m^2^	*n* = 41	*n* = 42	*n* = 41	*n* = 42	*n* = 40
Mean (SD)	23.7 (3.2)	23.9 (3.6)	23.1 (2.8)	24.7 (4.4)	24.5 (3.6)
Primary disease					
Diabetic nephropathy	16 (39.0)	12 (28.6)	17 (41.5)	10 (23.8)	20 (48.8)
Chronic glomerulonephritis	17 (41.5)	14 (33.3)	15 (36.6)	17 (40.5)	11 (26.8)
Nephrosclerosis	2 (4.9)	5 (11.9)	5 (12.2)	10 (23.8)	3 (7.3)
Polycystic kidney	1 (2.4)	4 (9.5)	2 (4.9)	3 (7.1)	1 (2.4)
Chronic pyelonephritis	0	1 (2.4)	0	0	0
Other	5 (12.2)	6 (14.3)	2 (4.9)	2 (4.8)	6 (14.6)
Phosphate binder before first washout					
Calcium carbonate	21 (51.2)	25 (59.5)	28 (68.3)	22 (52.4)	29 (70.7)
Sevelamer hydrochloride	9 (22.0)	7 (16.7)	11 (26.8)	5 (11.9)	5 (12.2)
Lanthanum carbonate	25 (61.0)	26 (61.9)	25 (61.0)	27 (64.3)	22 (53.7)
Bixalomer	5 (12.2)	1 (2.4)	6 (14.6)	6 (14.3)	0
Sucroferric oxyhydroxide	2 (4.9)	4 (9.5)	4 (9.8)	5 (11.9)	4 (9.8)
Ferric citrate hydrate	7 (17.1)	11 (26.2)	12 (29.3)	13 (31.0)	10 (24.4)
Phosphorus at enrollment, mg/dl					
Mean (SD)	7.5 (1.0)	7.3 (0.8)	7.5 (0.8)	7.3 (1.0)	7.4 (0.8)
≥6.1 to ≤8.0	32 (78.0)	33 (78.6)	32 (78.0)	32 (76.2)	32 (78.0)
≥8.1 to <10.0	9 (22.0)	9 (21.4)	9 (22.0)	10 (23.8)	9 (22.0)
Phosphorus at baseline, mg/dl					
Mean (SD)	7.6 (1.3)	7.5 (1.1)	8.1 (1.1)	7.7 (1.4)	7.4 (1.1)
≤6.0	6 (14.6)	2 (4.8)	2 (4.9)	4 (9.5)	6 (14.6)
≥6.1 to ≤8.0	20 (48.8)	27 (64.3)	18 (43.9)	23 (54.8)	23 (56.1)
≥8.1 to <10.0	14 (34.1)	12 (28.6)	19 (46.3)	12 (28.6)	12 (29.3)
≥10.0	1 (2.4)	1 (2.4)	2 (4.9)	3 (7.1)	0
Vitamin D	3 (80.5)	36 (85.7)	33 (80.5)	38 (90.5)	30 (73.2)
Calcium mimetics	20 (48.8)	23 (54.8)	13 (31.7)	25 (59.5)	14 (34.1)

BID, twice daily; max, maximum; min, minimum.

Data in the table are presented as *n* (%) unless otherwise stated. In the 30-mg dose-titration group, the number of patients was lower for day 1 measurements than other measurements because some subjects missed the day 1 measurements.

### Primary and Secondary End Points

The mean (SD) changes from baseline at week 6 (last observation carried forward) in serum P level were 0.6 (1.6) mg/dl in the placebo group and −0.9 (1.7) mg/dl, −1.4 (1.5) mg/dl, and −1.9 (1.2) mg/dl, in the tenapanor 5-mg BID, 10-mg BID, and 30-mg BID groups, respectively; in the dose 30-mg BID dose-titration group, the mean change in serum P level was −2.0 (1.1) mg/dl (Table 2). In all fixed-dose groups, tenapanor significantly decreased the serum P level from baseline compared with placebo (*P* < 0.001, for each dose). The same was observed in the 30-mg BID dose-titration group (*P* < 0.001). The serum P level decreases were more marked with increasing doses of tenapanor.

**Table 2:** Change from baseline in serum phosphorus level by group at week 6 (LOCF)

Parameter, unit	Placebo	Tenapanor (BID)			
		5 mg	10 mg	30 mg	30 mg dose-titration
*N*	41	42	41	42	41
Phosphorus (mg/dl)					
Mean (SD)	0.6 (1.6)	−0.9 (1.7)	−1.4 (1.5)	−1.9 (1.2)	−2.0 (1.1)
Median (min, max)	0.4 (−2.1, 4.9)	−1.0 (−4.8, 5.2)	−1.5 (−4.7, 2.8)	−2.1 (−4.4, 0.3)	−2.0 (−4.2, 0.6)
Difference from placebo		−1.6	−2.0	−2.6	−2.6
95% CI		[−2.3, −0.9]	[−2. 7, −1.3]	[−3.2, −2.0]	[−3.2, −2.0]
*P* value		< 0.001	< 0.001	< 0.001	< 0.001
Guideline achievement rate of serum phosphorus (≤6.0 mg/dl) at week 6, %	12.2	40.5	43.9	66.7	70.7

BID, twice daily; LOCF, last observation carried forward; max, maximum; min, minimum.

A Williams’ test was used for comparisons between the tenapanor fixed-dose groups and the placebo group. A *t* test was used for comparison between the tenapanor 30-mg BID dose-titration group and the placebo group.

**Figure 3:**
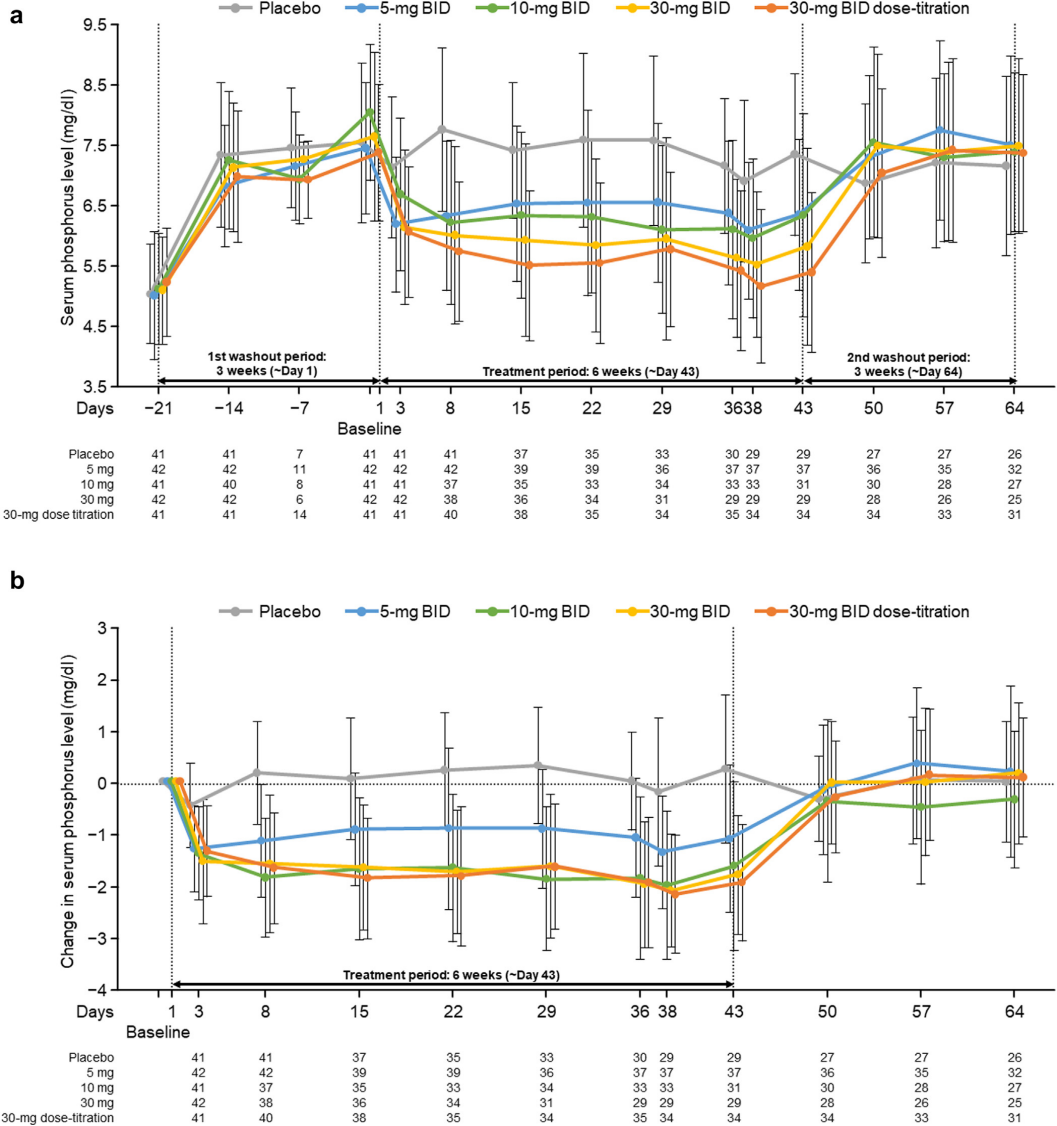
Mean (±SD) serum phosphorus level by group. (a) Transition from previous examination to the end of study drug administration. (b) Change from baseline from week 0 (day 3) to the end of study drug administration. Yellow-shaded area indicates guideline-recommended serum phosphorus level (≤6.0 mg/dl). BID, twice daily.

Regarding the transition of the mean serum P level by treatment group throughout the study, decreases in serum P in the tenapanor groups were observed from week 1 compared with placebo. These decreases generally remained constant during the 6 weeks of treatment (Figure 3a). Once the tenapanor administration was completed (week 6), serum P levels in all tenapanor groups returned to near baseline level by week 7.

Figure 3b illustrates the changes in mean serum P level by group from baseline between week 0 and the end of study drug administration (week 6). Within each tenapanor group, the mean change in the serum P level remained broadly constant from the beginning of the treatment period until its end. Greater mean changes were observed with increasing tenapanor dose.

**Figure 4:**
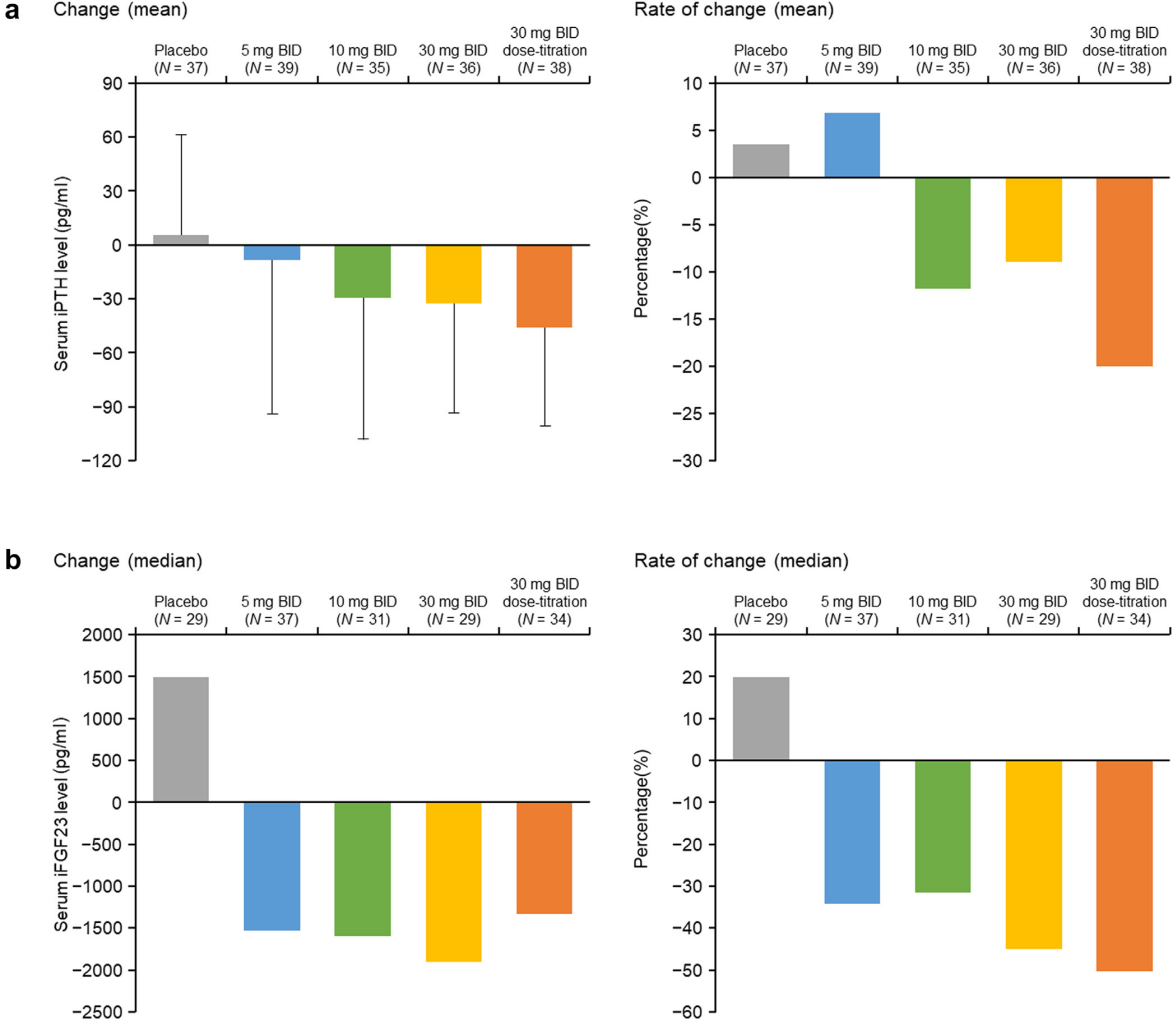
Mean changes and rates of changes of serum iPTH levels (a) and median of iFGF23 levels (b) by treatment group at the end of the study. BID, twice daily; iFGF23, intact fibroblast growth factor 23; LOCF, last observation carried forward; iPTH, intact parathyroid hormone.

Serum P target achievement rates were greater in the tenapanor 30-mg BID and 30-mg BID dose-titration groups compared with placebo and other tenapanor groups (Table 2). At week 6 (last observation carried forward), no significant mean (SD) changes in corrected serum Ca were observed in any group. The numerical (real) values of intact PTH (Figure 4a) and iFGF23 (Figure 4b) decreased in the tenapanor groups, and bone turnover markers (bone-specific alkaline phosphatase, osteocalcin, total N-terminal propeptide of type I collagen, and tartrate-resistant acid phosphatase 5b) remained mostly unchanged throughout the study (data not shown).

Medication adherence was 98.9%, 97.5%, 97.4%, 98.5%, and 97.9% for patients in the placebo and tenapanor 5-mg BID, 10-mg BID, 30-mg BID, and 30-mg BID dose-titration groups, respectively.

### Safety

Diarrhea was the most frequent AE by preferred term with incidences of 22.0% (9 of 41), 57.1% (24 of 42), 65.9% (27 of 41), 76.2% (32 of 42), and 70.7% (29 of 41) in the placebo, tenapanor 5-mg BID, 10-mg BID, 30-mg BID, and 30-mg BID dose-titration groups, respectively (Table 3). Serious AEs occurred in the tenapanor 5-mg BID group (*n* = 1, shunt stenosis), 10-mg BID group (*n* = 1, angina pectoris and *n* = 1, diverticulitis), 30-mg BID group (*n* = 1, arthritis), and the 30-mg BID dose-titration group (*n* = 1, shunt occlusion). None occurred with placebo. One case (2.4%) of severe diverticulitis classified as a serious AE occurred in the tenapanor 10-mg BID group and was considered by the investigator to be related to tenapanor.

**Table 3:** Summary of AEs and drug-related AEs by PT with an incidence >5%

PT	Tenapanor (BID)				
	Placebo	5 mg	10 mg	30 mg	30 mg dose-titration
*N*	41	42	41	42	41
AEs	21 (51.2)	33 (78.6)	32 (78.0)	36 (85.7)	33 (80.5)
Diarrhea	9 (22.0)	24 (57.1)	27 (65.9)	32 (76.2)	29 (70.7)
Nasopharyngitis	3 (7.3)	3 (7.1)	4 (9.8)	4 (9.5)	6 (14.6)
Arthralgia	3 (7.3)	0	0	1 (2.4)	0
Drug-related AEs					
Patients with any drug-related AE	7 (17.1)	22 (52.4)	28 (68.3)	32 (76.2)	28 (68.3)
Diarrhea	4 (9.8)	21 (50.0)	27 (65.9)	32 (76.2)	27 (65.9)

AE, adverse event; BID, twice daily; PT, preferred term.

Data in the table are presented as *n* (%).

The incidence of any drug-related AE in the placebo group was 17.1% (7 of 41), and that in the tenapanor 5-mg BID, 10-mg BID, 30-mg BID, and 30-mg BID dose-titration groups was 52.4% (22 of 42), 68.3% (28 of 41), 76.2% (32 of 42), and 68.3% (28 of 41), respectively. Diarrhea was the most frequent drug-related AE with incidences of 9.8% (4 of 41), 50.0% (21 of 42), 65.9% (27 of 41), 76.2% (32 of 42), and 65.9% (27 of 41) in the placebo and tenapanor 5-mg BID, 10-mg BID, 30-mg BID, and 30-mg BID dose-titration groups, respectively (Table 3).

In the 5-mg BID group, all cases of diarrhea were mild. In the tenapanor 10-mg BID and 30-mg BID fixed-dose groups, diarrhea was either mild or moderate. There were no cases of severe diarrhea in any of the tenapanor groups.

No clinically relevant alterations were noted in vital signs, laboratory values, or electrocardiogram parameters. There were no deaths during the study.

**Figure 5:**
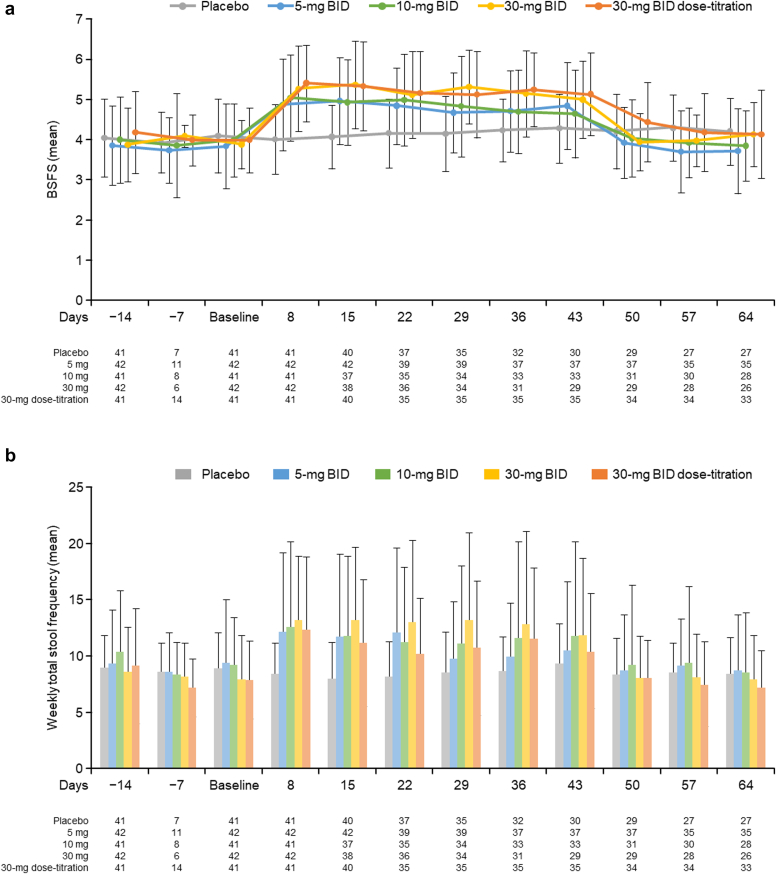
Changes in average BSFS scores (a) and stool frequency (b) per week. BID, twice daily; BSFS, Bristol Stool Form Scale.

Figure 5a illustrates the changes in average BSFS scores, and Figure 5b illustrates the defecation frequency per week by group. The mean change in the BSFS score was approximately 1 point in the tenapanor groups throughout the treatment period. The mean changes in BSFS scores were similar in the tenapanor 5-mg BID group and the tenapanor 10-mg BID group. The BSFS scores were numerically slightly higher in the tenapanor 30-mg BID group and the 30-mg BID dose-titration group. These changes remained broadly consistent within the tenapanor groups. After administration of tenapanor ceased, the scores returned to near baseline levels.

## Discussion

In this phase 2 clinical trial, we evaluated the efficacy and safety of tenapanor in Japanese patients with hyperphosphatemia undergoing hemodialysis. We found that, compared with placebo, tenapanor significantly reduced the serum P level from baseline in each dose group (*P* < 0.001). A positive dose-response relationship was observed for the serum P level-lowering effect of tenapanor. As expected, diarrhea was the most common AE, with an incidence of 57.1% to 76.2% in the tenapanor 5-mg, 10-mg, and 30-mg groups, compared with an incidence of 22% in the placebo group. The frequency of diarrhea seemed to increase in a dose-dependent manner. After the tenapanor administration ceased, serum P levels returned to near baseline levels. These findings confirm that the serum P-lowering effect was a result of the administration of tenapanor. In addition, a dose-response relationship was observed in both the guideline achievement rate of serum P, which increased from 40.5% to 70.7% in the tenapanor fixed-dose groups, and the serum P level-lowering effect.

A decrease in iFGF23 was observed in each tenapanor group. FGF23, along with PTH, contributes to the modulation of tubular reabsorption of phosphate. As iFGF23 is located upstream of the control of serum P level in humans,^24^ it is suggested that this decrease in iFGF23 by tenapanor may result from a regulatory feedback mechanism triggered by the reduction of the serum P level.

In this study, the most common AE and drug-related AE in each tenapanor group was diarrhea. Because the incidence of diarrhea increased with increasing tenapanor dose, diarrhea may have resulted from a dose-response effect during tenapanor treatment. Although diarrhea occurred in >70% of patients in the 30-mg BID group, only 20% of patients in that group discontinued tenapanor treatment. Diarrhea occurred relatively frequently in the 30-mg BID dose-titration group (70.7%); however, 71.4% (25 of 35) of patients did not require a dose adjustment during the treatment period and completed the study at the 30-mg dose. Among the tenapanor fixed-dose groups, the 5-mg BID dose group had the lowest incidence of diarrhea compared with the other active groups, and all cases were mild in severity. In the tenapanor 10-mg BID and 30-mg BID fixed-dose groups, there was some tolerability as all cases of diarrhea were either mild or moderate, with moderate cases accounting for approximately 10% and no cases of severe diarrhea. Although diarrhea occurred frequently in the tenapanor group, drug adherence was approximately 98% in all groups throughout the study period. These findings indicate that diarrhea was generally well tolerated for most patients receiving even the highest tenapanor dose and that it did not significantly interfere with administration of the study drug. The incidence of diarrhea reported in the 30-mg dose-titration group of the US phase 3 verification study was 47.9%,^21^ and the incidence of drug-related diarrhea (65.9%) was higher in this study. These differences could be explained by the differences in regions and studied populations; a direct comparison of the present results with those of the phase 3 study^21^ is not possible. Aside from diarrhea, there were no AEs judged to have a causal relationship with the investigational drug with an incidence rate exceeding 5%, which was consistent with previous clinical studies of tenapanor monotherapy conducted in the United States.^21^^,^^25^

The discontinuation rate was 36.6% in the placebo group and 23.8%, 34.1%, 40.5%, and 24.4% in the tenapanor 5-mg BID, 10-mg BID, 30-mg BID, and 30-mg BID dose-titration groups, respectively. Although only 8 patients discontinued because of AEs during the treatment period, 17 other patients discontinued owing to patient request for withdrawal during this period in the tenapanor groups. Nevertheless, it is possible that diarrhea could have been the underlying cause for discontinuing the study among these 17 patients. Although the incidence of diarrhea was similar between the 30-mg BID group and the 30-mg BID dose-titration group, in the latter, it is possible that the discontinuation rate during the treatment period was suppressed because the dose of tenapanor could be reduced.

The major pathway, accounting for approximately 50% of P absorption in the intestinal tract of rodents, is active phosphate absorption in the small intestine by sodium-dependent phosphate co-transporter type 2b (NPT-IIb or SLC34A2). The development of a drug for hyperphosphatemia targeting NPT-IIb was previously underway but was discontinued because of a lack of serum P level-lowering effect.^26^ The only drugs for hyperphosphatemia currently available are phosphate binders that physically bind to P in the intestinal tract, resulting in increased P excretion.^15^^,^^27^^,^^28^ Tenapanor has a novel mechanism of action; it acts on the intestinal sodium-hydrogen exchanger 3 transporter and inhibits passive transport of P through the intestinal epithelial cell gap.^19^^,^^29^ The present results confirm this effect by revealing a significant decrease in serum P level in all tenapanor groups compared with placebo.

This study had several limitations, such as a short treatment period of 6 weeks. We would like to conduct a tenapanor administration study to confirm its long-term efficacy and safety. In addition, we did not evaluate doses <5 mg or >30 mg in this study. Only Japanese patients were included, which may also limit the generalizability of the results to other ethnicities. Finally, patient dietary records were not collected; therefore, we cannot rule out the possibility that patients’ diets may have affected their serum P level.

In conclusion, tenapanor significantly decreased the serum P levels from baseline to week 6 compared with the placebo group, even at the minimum dose of tenapanor (5-mg BID dose group), in Japanese patients with hyperphosphatemia undergoing hemodialysis. There was a dose-response relationship between the efficacy of tenapanor and its serum P-lowering effect. There was a high rate of achievement of guideline levels for serum P levels with tenapanor compared with placebo. The most common AEs and drug-related AEs were gastrointestinal AEs, among which mild diarrhea was the most frequent. On the basis of these findings, the recommended starting dose in Japan is tenapanor 5 mg BID, which may minimize safety risks while ensuring P-lowering efficacy. These data suggest that tenapanor can be a first-in-class therapeutic agent with a different mechanism of action from existing P-adsorbing agents.

## Disclosure

MI reports receiving personal fees from Kyowa Kirin Co., Ltd., Bayer Japan, Daiichi Sankyo Co., Ltd., Chugai Pharmaceutical Co., Ltd., Pfizer Co. Ltd., Ono Pharmaceutical Co., Ltd., Torii Pharmaceutical Co. Ltd., and Kissei Co. Ltd., during the conduct of the study. MF reports receiving personal fees from Kyowa Kirin Co., Ltd., Ono Pharmaceutical Co. Ltd., Torii Pharmaceutical Co. Ltd., and Kissei Pharmaceutical Co. Ltd., and grants from Bayer Yakuhin Ltd., during the conduct of the study. TA reports receiving personal fees from Kyowa Kirin Co., Ltd., during the conduct of the study, and personal fees from Astellas, Bayer Yakuhin Ltd., Kissei Pharmaceutical Co. Ltd., Ono Pharmaceutical Co. Ltd., Fuso Pharmaceutical Industries Ltd., Torii Pharmaceutical Co. Ltd., GlaxoSmithKline, JT Pharmaceuticals, Nipro Corporation, Otsuka, Sanwa Chemical, and Chugai Pharmaceutical Co. Ltd., outside of the submitted work. YU, KI, and HK are employees of Kyowa Kirin Co., Ltd.

## Supplementary Material

**Supplementary Methods.**

**Prohibited and Allowed Concomitant Medications**. This section includes the details regarding prohibited and allowed concomitant medications.

**Other Exclusion Criteria**. This section lists the additional exclusion criteria that are not listed in the main manuscript text.

**CONSORT Statement**
