# Corrigendum to Inaba M, Une Y, Ikejiri K, et al. “Dose-Response of Tenapanor in Patients With Hyperphosphatemia Undergoing Hemodialysis in Japan—A Phase 2 Randomized Trial.” *Kidney Int Rep*. 2022;7:177–188

**DOI:** 10.1016/j.ekir.2022.06.008

**Published:** 2022-07-05

The authors regret that in the above-stated article, the number and percentage of people taking vitamin D in the placebo group was incorrectly listed as “3 (80.5)” in Table 1. The correct number and percentage are “33 (80.5).”

The authors would like to apologize for any inconvenience caused.

