# Supplementary material for: Dose-Response of Tenapanor in Patients With Hyperphosphatemia Undergoing Hemodialysis in Japan—A Phase 2 Randomized Trial
Source: Kidney Int Rep. 2021 Nov 24;7(2):177–88. doi: 10.1016/j.ekir.2021.11.008 (PMC8820999; doi:10.1016/j.ekir.2021.11.008)
Supplement: Supplementary File (PDF) [file mmc1.pdf]

Supplementary Material

Supplementary Methods

Prohibited and Allowed Concomitant Medications

The concomitant use of the following medications and therapies was prohibited: phosphate binders; aluminum preparations, niceritrol, nicotinamide, colestimide, and cholestyramine; other supplements for phosphorus adsorption (such as calcium acetate and eggshell calcium); and peritoneal dialysis. Active vitamin D preparations, or their derivatives, and calcimimetics could be used if used at the same dose for at least 4 weeks before the screening examination. Use of calcium preparations for calcium supplementation was allowed during the study. For corrected serum calcium levels > 11.0 mg/dL after the start of study treatment, active vitamin D preparations or their derivatives could be reduced or discontinued. Use of new vitamin D preparations or their derivatives or an increase in their dose was allowed if the corrected serum calcium levels was ≤ 7.5 mg/dL or the patient presented clinical symptoms presumed to be due to hypocalcemia.

Other Exclusion Criteria

Other exclusion criteria at pre-enrollment were heart disease (including congestive heart failure, defined as New York Heart Association cardiac functional classification of class III or IV, and cardiovascular disease requiring hospitalization, such as myocardial infarction) or hepatic impairment (including aspartate aminotransferase/alanine aminotransferase ≥ 100 U/L during the first washout period and cirrhosis); cerebrovascular disease (such as cerebral infarction and hemorrhage) or cardiovascular disease (such as myocardial infarction and unstable angina) requiring hospitalization; uncontrollable hypertension or diabetes; newly scheduled for living donor kidney transplant; change in the mode of dialysis, home hemodialysis, or change in the

dialysis center (relocation to another hospital/clinic) during the study period; diagnosis of malignancy or newly discovered concurrent malignancy; tested positive for human immunodeficiency virus or human T-cell leukemia virus type 1; serious drug allergies, such as anaphylactic shock, after pre-enrollment or any evidence of alcohol dependence, illicit drug use, severe mental illness, or drug abuse or addiction; received other study drugs or tenapanor before enrollment; female patients found to be pregnant or who desired to bear children during the study period; and newly discovered medical or other conditions that, in the opinion of the investigator or subinvestigator, precluded participation in the study.

CONSORT 2010 checklist of information to include when reporting a randomised trial*

Section/Topic	Item No	Checklist item	Reported on page No
**Title and abstract**			
	1a	Identification as a randomised trial in the title	1
	1b	Structured summary of trial design, methods, results, and conclusions (for specific guidance see CONSORT for abstracts)	3
**Introduction**			
Background and objectives	2a	Scientific background and explanation of rationale	4, 5
	2b	Specific objectives or hypotheses	5
**Methods**			
Trial design	3a	Description of trial design (such as parallel, factorial) including allocation ratio	6, 7
	3b	Important changes to methods after trial commencement (such as eligibility criteria), with reasons	N/A
Participants	4a	Eligibility criteria for participants	6-8
	4b	Settings and locations where the data were collected	6
Interventions	5	The interventions for each group with sufficient details to allow replication, including how and when they were actually administered	6, 7
Outcomes	6a	Completely defined pre-specified primary and secondary outcome measures, including how and when they were assessed	8
	6b	Any changes to trial outcomes after the trial commenced, with reasons	N/A
Sample size	7a	How sample size was determined	9
	7b	When applicable, explanation of any interim analyses and stopping guidelines	N/A

Randomisation:			
Sequence generation	8a	Method used to generate the random allocation sequence	6, 7
	8b	Type of randomisation; details of any restriction (such as blocking and block size)	6
Allocation concealment mechanism	9	Mechanism used to implement the random allocation sequence (such as sequentially numbered containers), describing any steps taken to conceal the sequence until interventions were assigned	6
Implementation	10	Who generated the random allocation sequence, who enrolled participants, and who assigned participants to interventions	6
Blinding	11a	If done, who was blinded after assignment to interventions (for example, participants, care providers, those assessing outcomes) and how	6
	11b	If relevant, description of the similarity of interventions	N/A
Statistical methods	12a	Statistical methods used to compare groups for primary and secondary outcomes	9
	12b	Methods for additional analyses, such as subgroup analyses and adjusted analyses	8
**Results**			
Participant flow (a diagram is strongly recommended)	13a	For each group, the numbers of participants who were randomly assigned, received intended treatment, and were analysed for the primary outcome	10
	13b	For each group, losses and exclusions after randomisation, together with reasons	10
Recruitment	14a	Dates defining the periods of recruitment and follow-up	6
	14b	Why the trial ended or was stopped	N/A
Baseline data	15	A table showing baseline demographic and clinical characteristics for each group	22-24
Numbers analysed	16	For each group, number of participants (denominator) included in each analysis and whether the analysis was by original assigned groups	10

Outcomes and estimation	17a	For each primary and secondary outcome, results for each group, and the estimated effect size and its precision (such as 95% confidence interval)	11, 12
	17b	For binary outcomes, presentation of both absolute and relative effect sizes is recommended	N/A
Ancillary analyses	18	Results of any other analyses performed, including subgroup analyses and adjusted analyses, distinguishing pre-specified from exploratory	12, 13
Harms	19	All important harms or unintended effects in each group (for specific guidance see CONSORT for harms)	12, 13
**Discussion**			
Limitations	20	Trial limitations, addressing sources of potential bias, imprecision, and, if relevant, multiplicity of analyses	15
Generalisability	21	Generalisability (external validity, applicability) of the trial findings	15
Interpretation	22	Interpretation consistent with results, balancing benefits and harms, and considering other relevant evidence	13-15
**Other information**			
Registration	23	Registration number and name of trial registry	6
Protocol	24	Where the full trial protocol can be accessed, if available	N/A
Funding	25	Sources of funding and other support (such as supply of drugs), role of funders	17

*We strongly recommend reading this statement in conjunction with the CONSORT 2010 Explanation and Elaboration for important clarifications on all the items. If relevant, we also recommend reading CONSORT extensions for cluster randomised trials, non-inferiority and equivalence trials, non-pharmacological treatments, herbal interventions, and pragmatic trials. Additional extensions are forthcoming: for those and for up to date references relevant to this checklist, see www.consort-statement.org.
